# Electrolyte Optimization Strategy: Enabling Stable and Eco-Friendly Zinc Adaptive Interfacial Layer in Zinc Ion Batteries

**DOI:** 10.3390/molecules29040874

**Published:** 2024-02-16

**Authors:** Bozhong Cao, Chunyan Xu, Bingchun Jiang, Biao Jin, Jincheng Zhang, Lei Ling, Yusheng Lu, Tianyu Zou, Tong Zhang

**Affiliations:** 1 College of Mechanical and Electrical Engineering, Guangdong University of Science and Technology, Dongguan 523000, China; caobaizhong@gdust.edu.cn (B.C.); jiangbingchun@gdust.edu.cn (B.J.); jinbiao@gdust.edu.cn (B.J.); zhangjincheng2@gdust.edu.cn (J.Z.); linglei1@gdust.edu.cn (L.L.); luyusheng@gdust.edu.cn (Y.L.); zoutianyu@gdust.edu.cn (T.Z.); 2 Institute for Interdisciplinary Quantum Information Technology, Jilin Engineering Normal University, Changchun 130052, China; xcy@jlenu.edu.cn

**Keywords:** aqueous zinc ion batteries, electrolyte optimization, stable interfacial layer, zinc dendrite growth

## Abstract

Aqueous zinc ion batteries (AZIBs) have emerged as a promising battery technology due to their excellent safety, high capacity, low cost, and eco-friendliness. However, the cycle life of AZIBs is limited by severe side reactions and zinc dendrite growth on the zinc electrode surface, hindering large-scale application. Here, an electrolyte optimization strategy utilizing the simplest dipeptide glycylglycine (Gly-Gly) additive is first proposed. Theoretical calculations and spectral analysis revealed that, due to the strong interaction between the amino group and Zn atoms, Gly-Gly preferentially adsorbs on zinc’s surface, constructing a stable and adaptive interfacial layer that inhibits zinc side reactions and dendrite growth. Furthermore, Gly-Gly can regulate zinc ion solvation, leading to a deposition mode shift from dendritic to lamellar and limiting two-dimensional dendrite diffusion. The symmetric cell with the addition of a 20 g/L Gly-Gly additive exhibits a cycle life of up to 1100 h. Under a high current density of 10 mA cm^−2^, a cycle life of 750 cycles further demonstrates the reliable adaptability of the interfacial layer. This work highlights the potential of Gly-Gly as a promising solution for improving the performance of AZIBs.

## 1. Introduction

The need to construct corresponding large-scale energy storage systems is being driven by the world’s installed renewable power capacity expanding at an accelerated rate. However, the development of traditional lithium-ion batteries is limited by their organic electrolyte safety, high cost, and the shortage of lithium metal resources [1,2,3,4]. These pressures can be successfully relieved by applying an aqueous electrolyte in battery systems. Since the electrochemical intercalation/deintercalation of Na^+^, K^+^, Mg^2+^, and Zn^2+^ in aqueous electrolytes offers a high degree of security, abundant material resources, and environmental friendliness, it has, therefore, received a lot of attention. Among them, zinc ion batteries (ZIBs) have attracted great attention due to their inherent security, high specific capacity (820 mAh g^−1^), and low charge/discharge potential. However, the dendrite growth occurring at the anode–electrolyte interface (AEI) and water-induced side reactions are still the main challenges to the practical application of ZIBs. [5,6,7]. These side reactions involve the reaction of zinc metal with water molecules in the zinc sulfate electrolyte, leading to the generation of hydrogen (HER) and insoluble non-electrochemically active byproducts (Zn_4_SO_4_[OH_6_]-xH_2_O). These reactions result in the uneven deposition of zinc ions on the electrodes, accelerated corrosion, and the passivation of the zinc metal, which lead to issues such as dendrites puncturing the separator and gas build-up in the battery shell [8,9,10,11,12]. These problems ultimately impact the reversibility of the charge and discharge processes in AZIBs, thereby affecting their cycle life.

Several improvement methods have been proposed to address the various challenges faced by zinc-ion batteries, such as electrolyte optimization, negative electrode modification, and positive electrode structural design [13,14,15,16,17,18,19,20,21,22,23,24,25]. Among these methods, electrolyte optimization through the addition of additives has been proven to be a simple, effective, and cost-efficient approach [26,27]. Research on electrolyte additives can be categorized into two major directions. Firstly, the addition of soluble inorganic metal salts or organic substances to the electrolyte can regulate the solvation structure of zinc ions, inhibit side reactions, and control the deposition process of zinc ions. These additives aim to overcome problems such as the dendrite puncturing of the separator and gas build-up in the battery shell, ultimately prolonging the cycle life of AZIBs. For instance, Cao et al. demonstrated the use of the SnCl_2_ additive in ZnCl_2_ electrolytes to regulate the solvation structure of zinc ions and create a zinc-friendly Zn_5_(OH)_8_Cl_2_-H_2_O solid electrolyte interphase (SEI) on zinc’s surface [28]. Wang et al. employed the Silk peptide as an additive in the zinc sulfate electrolyte, utilizing the abundant polar groups (-COOH and -NH_2_) to modulate the solvation and interfacial behavior of zinc and change the dendritic growth mode [29]. Lu et al. successfully inhibited dendrite formation and side reactions by introducing positively charged arginic acid additives into zinc sulfate electrolytes [30]. Additionally, numerous research reports have indicated that many organic molecules can selectively adsorb on the surface of zinc foils, acting as protective layers to isolate zinc from water and alter the solvation structure of zinc ions [31,32,33,34,35,36,37,38].

This paper presents a strategy for optimizing zinc sulfate electrolytes through the addition of glycylglycine (Gly-Gly), a dipeptide molecule with abundant polar groups (-COOH and -NH_2_). Gly-Gly acts as an electrolyte additive and plays a crucial role in regulating the solvation structure of zinc ions. Additionally, it forms a stable interfacial layer on the zinc electrode surface, effectively isolating the electrode from water molecules. Consequently, the deposition structure of zinc is altered, resulting in the improved longevity of AZIBs (aqueous zinc-ion batteries). Experiments have demonstrated that at a Gly-Gly concentration of 20 g/L, Zn-Zn symmetric cells can achieve a cycle life of 1100 h at 1 mA cm^−2^ and 1 mAh cm^−2^. These cells also exhibit stable cycling behavior for 750 cycles at a high current density of 10 mA cm^−2^ and 1 mAh cm^−2^. In addition, the adaptive Zn–electrolyte interface formed using Gly-Gly ensures the stability and reversibility of AZIBs, as evidenced by a Coulombic efficiency (CE) of 99.6% in Ti-Zn half-cells with a high specific capacity of 206 mAh g^−1^ after 100 cycles at 200 mA g^−1^ in the Zn//MnO_2_ full battery.

## 2. Results and Discussion

**Figure 1:**
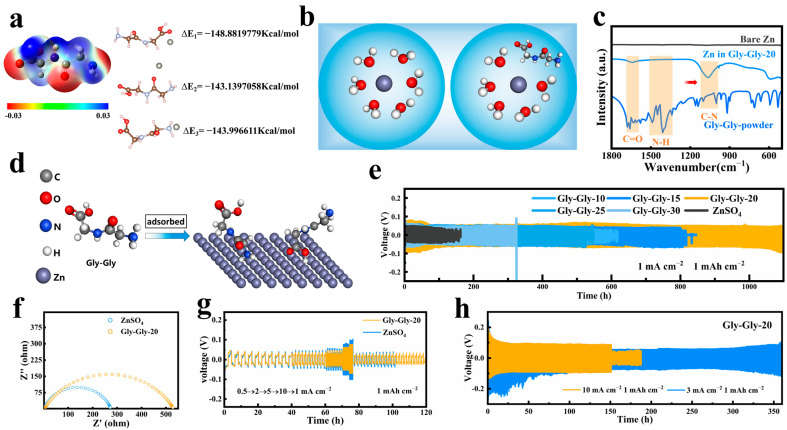
(**a**) DFT calculations of the binding energies (ΔE_1_, ΔE_2_, and ΔE_3_) between Zn^2+^ and the -COOH, -NH_2_, and -CO-NH- groups, respectively, on Gly-Gly chains. (**b**) Gly-Gly modulates the solvated structure of Zn^2+^. (**c**) The FTIR spectrum of bare Zn, Zn soaked in a Gly-Gly solution and pure Gly-Gly. (**d**) The adsorption of Gly-Gly on the surface of Zn. (**e**) Cycling performance of Zn-Zn symmetric cells with different concentrations of Gly-Gly additives at a current density of 1 mA cm^−2^ and 1 mAh cm^−2^. (**f**) Electrochemical impedance spectroscopy (EIS) of Zn-Zn symmetric cells before cycling in ZnSO_4_ electrolytes with and without Gly-Gly additives. (**g**) The rate performance of Zn-Zn symmetric cells with and without Gly-Gly additives. (**h**) Cycling performance of Zn-Zn symmetric cells with Gly-Gly additives at different current densities.

We utilized two different electrolytes in our experiments: a standard electrolyte consisting of 2M of ZnSO_4_ and an optimized electrolyte (Gly-Gly-20) composed of 2M of ZnSO_4_ with an additive of 20 g/L of Gly-Gly. Additionally, we prepared electrolytes with different concentrations of Gly-Gly (Gly-Gly-10/15/25/30). Firstly, the stability of Gly-Gly in the zinc sulfate electrolyte was investigated, and it was found that upon the addition of Gly-Gly, there was a slight decrease in the pH value (Figure S1 Supplementary Materials). The increase in the Gly-Gly concentration did not significantly impact the pH value, indicating that only a small amount of Gly-Gly underwent hydrolysis in the electrolyte, leading to the generation of glycine and the consumption of water molecules. It was determined that Gly-Gly exhibited superior ionization rather than hydrolysis reactions, resulting in an observed decrease in the pH value [32]. Furthermore, the interaction between Gly-Gly and Zn ions was examined through the density-functional theory (DFT). Electrostatic potential mapping revealed a prominent charge distribution in the Gly-Gly molecule, particularly the negatively charged electrostatic potentials present in the amino and carboxyl groups (Figure 1a). This provided an enhanced electrostatic attraction between the Zn^2+^ and Gly-Gly molecules. The negatively charged groups also serve as nucleophilic sites that cooperate with Zn^2+^, thereby controlling the solvated structure of Zn^2+^ and influencing its nucleation and deposition. In addition, the calculated binding energies displayed higher values for the interactions of Zn ions with Gly-Gly carboxyl groups (−148.8819779 Kcal mol^−1^), amide groups (−143.1397058 Kcal mol^−1^), and amino groups (−143.996611 Kcal mol^−1^) compared to the binding energy with water (−42.70348002 Kcal mol^−1^). These elevated binding energies facilitate the exchange of water molecules from the solvated structure of zinc via Gly-Gly molecules (Figure 1b), thereby altering the solvated structure of zinc [35,39,40,41,42].

To investigate the interaction between Gly-Gly and zinc foil in zinc sulfate electrolytes, we immersed the zinc foil in ZnSO_4_ electrolytes with Gly-Gly-20 additives for five days. The FTIR spectra of immersed zinc foil exhibited similarities to those of Gly-Gly powder (Figure 1c), displaying prominent C-N peaks and C=O peaks at 1060 cm^−1^ and 1640 cm^−1^, respectively. The red shift of these peaks indicated a strong interaction between zinc and Gly-Gly. Interestingly, the addition of Gly-Gly resulted in the absence of N-H peaks on the zinc foil surface (1490 cm^−1^), which could be attributed to the chemisorption of Gly-Gly on the zinc’s surface (Figure 1d) [43]. Surface morphology analysis of the zinc foils was performed using SEM, as provided in Figure S2. Upon immersion in the ZnSO_4_ electrolyte, significant zinc dendrites appeared on the zinc foil surface. However, when immersed in the Gly-Gly-20 electrolyte, the zinc foil exhibited a laminar structure and appeared extremely flat. Notably, no significant zinc dendrites were observed at the 20 μm scale. These observations indicate that Gly-Gly effectively alters the deposition structure of zinc [43].

Next, we performed a constant current charge/discharge test to evaluate the effect of the Gly-Gly additive on the cycle life of the Zn-Zn symmetric cell at a current density of 1 mA cm^−2^ and areal capacity of 1 mAh cm^−2^. As shown in Figure 1e, the ZnSO_4_ electrolyte experienced a significant voltage drop after 150 h of cycling due to the penetration of zinc dendrites through the membrane, leading to the failure of the Zn-Zn symmetric cell. While the addition of Gly-Gly significantly prolonged the cycle life of Zn-Zn symmetric cells, when the concentration of Gly-Gly was reached at 20 g/L, the cycle life of Zn-Zn symmetric cells might reach this after about 1100 h. In addition, we also used ultra-high current density (3 mA cm^−2^ 1 mAh cm^−2^, 10 mA cm^−2^, and 1 mAh cm^−2^) to investigate the behavior of the cells under extreme conditions (Figure 1h). In general, the battery life was very short at high current densities due to Zn dendrite growth; however, the lifetime of the cells with Gly-Gly-20 reached 360 h at 3 mA cm^−2^ and 750 cycles at 10 mA cm^−2^. We also tested the rate performance of symmetric cells at different current densities, as shown in Figure 1g due to the presence of severe dendrite growth; the cells with bare ZnSO_4_ electrolyte easily became short-circuited after 100 h. This phenomenon was greatly alleviated by the addition of Gly-Gly. In summary, the introduction of Gly-Gly in the electrolyte could lead to a great improvement in the cycle life of AZIBs and showed good adaptability in ultra-high current conditions and rate tests. Remarkably, the introduction of Gly-Gly in the electrolyte led to an increased cell voltage hysteresis during the cycling test when compared to the pure ZnSO_4_ electrolyte. This could be attributed to the formation of an adaptive interfacial layer on the surface of the zinc electrode, which creates spatial potential resistance. In addition, the voltage hysteresis polarization of the zinc–zinc symmetric cell decreases and stabilizes after 72 h of cycling, which we attribute to the adaptive ability of Gly-Gly playing a role in guiding the uniform deposition of Zn^2+^ and thus reducing the spatial potential resistance of the interfacial layer. To investigate this phenomenon further, impedance tests were performed on the symmetric cells. The results before cycling demonstrated that the addition of the Gly-Gly additive caused an increase in the charge transfer impedance (Rct) of the symmetric cell in comparison to the pure ZnSO_4_ electrolyte (Figure 1f). This increase in Rct serves as further confirmation of the adsorption of Gly-Gly molecules onto zinc’s surface [44,45,46,47]. However, after 10 cycles (Figure S3), the Rct of both symmetric cells decreased, which was attributed to the electrochemical activation effect. Nevertheless, as the cycle proceeded, the symmetric cell with Gly-Gly-20 electrolyte showed a much lower Rct than that with the pure ZnSO_4_ electrolyte due to the smooth Zn deposited owing to the Gly-Gly. This also demonstrates that the side reactions of Zn anodes were effectively suppressed in the Gly-Gly-20 electrolyte. Collectively, these findings reinforce the notion that the incorporation of Gly-Gly enhances the overall performance, adaptability, and stability of the zinc-ion battery system.

**Figure 2:**
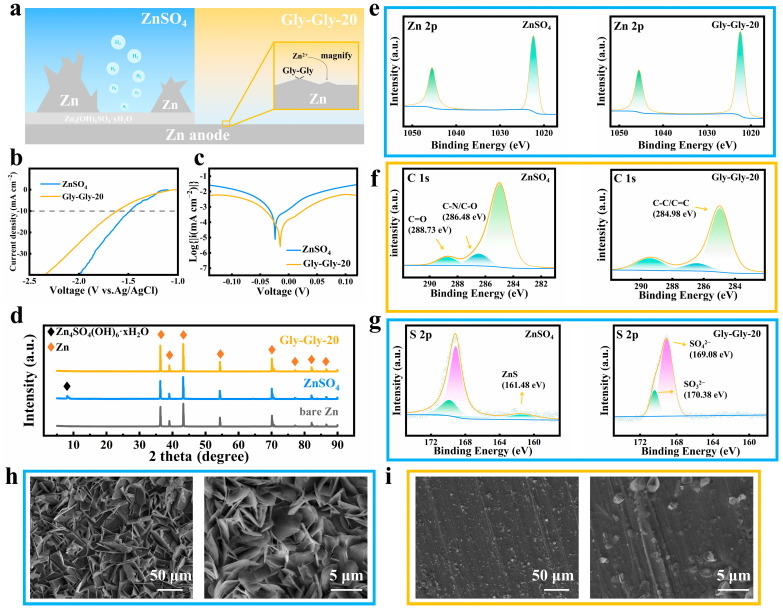
(**a**) Schematic illustration of Gly-Gly acts as the interfacial electrostatic shield on the Zn anode. (**b**) Hydrogen evolution reaction (HER) curves of ZnSO_4_ electrolytes with/without Gly-Gly. (**c**) The Tafel curves of ZnSO_4_ electrolytes with/without Gly-Gly. (**d**) X-ray diffraction (XRD) analysis of the zinc anode from symmetric cells after 20 h of cycling. X-ray photoelectron spectroscopy (XPS) spectra of (**e**) Zn 2p, (**f**) C 1s XPS, and (**g**) S 2p obtained from the zinc anode after 20 h of cycling without/with Gly-Gly. (**h**,**i**) Scanning electron microscopy (SEM) image of the zinc anode cycled for 20 h without/with Gly-Gly.

The occurrence of side reactions in zinc ion batteries often leads to battery failure. Figure 2a illustrates the hydrogen evolution reaction (HER) that takes place at the zinc electrode during the charging and discharging process. The resulting hydrogen causes the battery case to become clogged, eventually leading to a disconnection. To address this issue, Gly-Gly was introduced into the electrolyte. It was observed that Gly-Gly forms a stable and adaptable interfacial layer on the surface of the zinc electrode, effectively mitigating the HER. In order to investigate the inhibitory effect of Gly-Gly on the HER, an experiment was conducted using linear sweep voltammetry (LSV) at 1 mV s^−1^. To eliminate the interference of zinc deposition, the 2 M ZnSO_4_ electrolyte was replaced with a 1 M Na_2_SO_4_ electrolyte. The LSV curve obtained from this experiment, as shown in Figure 2b, reveals that the initial potential of the electrolyte with Gly-Gly additive was reduced by approximately 130 mV compared to the pristine ZnSO_4_ electrolyte when a current density of 10 mA cm^−2^ was applied. This reduction in potential indicates a suppression of the reaction between zinc and water. Furthermore, a Tafel curve analysis was performed simultaneously. The results, depicted in Figure 2c, show that the ZnSO_4_ electrolyte with the Gly-Gly additive exhibited an increase in the corrosion potential (from −0.024 V to −0.015 V) and a decrease in the corrosion current density (from 2.08 mA cm^−2^ to 0.604 mA cm^−2^) compared to the bare ZnSO_4_ electrolyte. This suggests that the corrosion of the zinc electrode was significantly inhibited. Overall, the introduction of Gly-Gly as an additive in the electrolyte of zinc ion batteries effectively mitigates the side reactions, such as the HER. It forms a stable interfacial layer on the zinc electrode that hinders the corrosion of zinc foils. These findings highlight the potential of Gly-Gly as a means to enhance the performance and lifespan of zinc ion batteries.

The formation of insoluble by-products (Zn_4_SO_4_(OH_6_)-xH_2_O) during side reactions had a negative impact on the zinc cycling process by causing the surface passivation of the zinc electrode. X-ray diffraction (XRD) characterization was performed on the zinc electrode that had been cycled in different electrolytes for 20 h, as shown in Figure 2d. A comparison was made between zinc foils cycled in ZnSO_4_ electrolytes both with and without the addition of the Gly-Gly additive. It was observed that the characteristic peaks of Zn_4_SO_4_[OH_6_]-xH_2_O diffraction, located at 8.12°, disappeared after the introduction of the Gly-Gly additive, indicating the effective inhibition of by-product formation due to the Gly-Gly. As to the X-ray photoelectron spectroscopy (XPS), the Zn2p peaks at 1045.58 eV and 1022.38 eV remained unchanged, as depicted in Figure 2e, indicating the preservation of the chemical environment suitable for zinc deposition in the electrolyte. Remarkably, the intensity of the C=O peak located at 288.73 eV significantly increased due to the presence of the -COOH group in Gly-Gly, indicating the notable adsorption of Gly-Gly at the zinc electrode (Figure 2f). Furthermore, the disappearance of the ZnS peak located at 161.48 eV provided additional evidence of Gly-Gly’s inhibition of the binding between S^2−^ ions and Zn^2+^, consequently reducing the generation of by-products (Figure 2g). The energy-dispersive X-ray spectroscopy (EDS) energy spectrum is depicted in Figure S4 and Table S1. Remarkably, sulfur (S) elements were reduced, indicating the effective inhibition of by-product formation by Gly-Gly, which is consistent with the results of XRD. These experimental observations demonstrate the effective role of Gly-Gly as an additive in inhibiting the formation of non-electrochemically active by-products, maintaining the proper chemical environment for zinc deposition, and preventing the binding of S^2−^ ions with Zn^2+^. Overall, the utilization of Gly-Gly contributes to the improved performance and integrity of zinc-ion batteries.

One of the main causes of cell failure is the growth of zinc dendrites on the zinc electrode. These dendrites can penetrate the membrane, leading to a short-circuit and significantly reducing the reversibility of the cell. Figure 2h illustrates the severe deterioration of the surface of the bare zinc foil in the ZnSO_4_ electrolyte after 20 h of cycling. This deterioration is caused by the accumulation of hexagonal nanosheets, also known as zinc dendrites. However, when the ZnSO_4_ electrolyte was supplemented with the Gly-Gly additive, the zinc electrode maintained a smooth surface (Figure 2i). This is attributed to the ability of Gly-Gly to immobilize the zinc electrode surface and produce an electrostatic shielding effect. This effect induces a uniform distribution of zinc ions [48]. Furthermore, as demonstrated in Figure S5, even after 800 h of cycling in the Gly-Gly-20 electrolyte, the surface of the zinc electrode remains smooth. This further confirms the inhibitory effect of the Gly-Gly-20 electrolyte on the two-dimensional growth of zinc dendrites [29,48].

**Figure 3:**
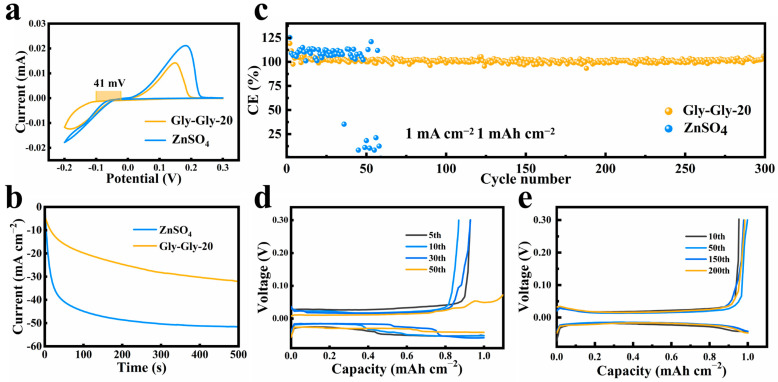
(**a**) Comparison of Zn stripping/plating behaviors in ZnSO_4_ electrolytes with and without Gly-Gly: cyclic voltammetry (CV) analysis. (**b**) Comparison of Zn behavior in electrolytes with/without Gly-Gly: Chronoamperometry (CAs). (**c**) Zn plating/stripping coulombic efficiencies (CEs) on Ti foil in electrolytes with/without Gly-Gly at 1 mA cm^−2^, 1 mAh cm^−2^: (**d**,**e**) Voltage profiles at different cycles of the Zn–Ti cell in ZnSO_4_ electrolytes with/without Gly-Gly at 1 mA cm^−2^, 1 mAh cm^−2^.

To investigate the synergistic effect of Gly-Gly on solvation and interfacial modulation, Ti-Zn half-cells were assembled to test the reversible behavior of zinc in the Gly-Gly-20 electrolyte. CV tests revealed that, compared to the bare ZnSO_4_ electrolyte, the zinc nucleation overpotential increased by 41 mV in the Gly-Gly-20 electrolyte (Figure 3a). This increase in overpotential led to a stronger nucleation drive, allowing for the formation of smaller Zn nuclei and promoting preferred nuclei orientation growth. Consequently, this restricted the random two-dimensional diffusion of Zn^2+^ and mitigated the “tip effect” [29,30,34,49]. These findings are also consistent with the results of the time amperometric curve test (Figure 3b). For the bare ZnSO_4_ electrolyte, the current density continuously increased when a negative overpotential of −150 mV was applied to the zinc–zinc symmetric cell. This indicates the presence of a continuous two-dimensional planar diffusion process, leading to zinc accumulation at the dendrite tips. However, in the Gly-Gly-20 electrolyte, the current density increase slowed down after the initial 50 s and gradually reached a plateau, suggesting a shift from the two-dimensional to three-dimensional diffusion of zinc ions, resulting in suppressed dendrite growth [37,48,50,51]. The coulombic efficiency (CE) is another important factor in evaluating cell performance (Figure 3c). The addition of Gly-Gly resulted in a high CE of 99.6% for the Ti-Zn half-cell, indicating excellent reversibility. By contrast, the bare ZnSO_4_ electrolyte showed significant fluctuations in CE and failed to reach the cutoff voltage after 50 cycles. The plating-stripping voltage profiles during different cycles of the cells are shown in Figure 3d,e. The initial voltage hysteresis of the bare ZnSO_4_ electrolyte is about 77.6 mV, which is higher than that of the Gly-Gly-20 (30.1 mV), demonstrating that the addition of Gly-Gly reduces the energy barrier for the nucleation/dissolution of Zn^2+^. These electrochemical results strongly demonstrate the effectiveness of the Gly-Gly additive, which can be primarily attributed to the formation of a stable and adaptive interfacial layer facilitated by the strong chemisorption of Gly-Gly molecules. This layer effectively inhibits severe side reactions and enables the long-term stable cycling of the zinc-ion battery [52,53,54]. Additionally, the extensive use of Gly-Gly in other fields presents advantages in terms of availability and environmental friendliness.

**Figure 4:**
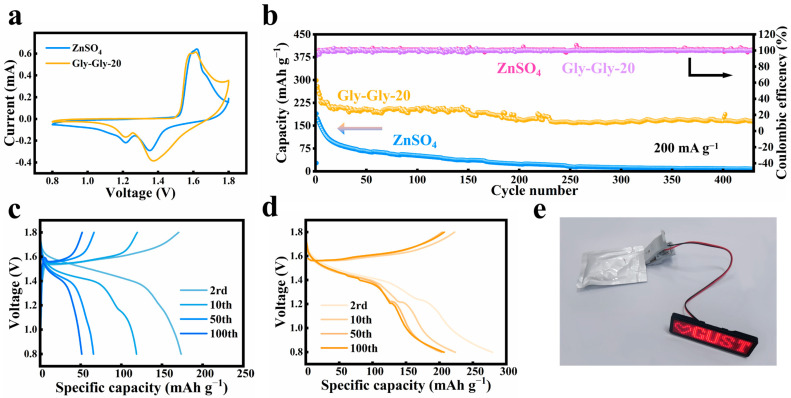
(**a**) CV characterization of MnO_2_//Zn full cells in electrolytes with and without Gly-Gly-20 (First Cycle, 0.5 mV s^−1^). (**b**) Investigation of long-term cycling stability of MnO_2_//Zn full cells in ZnSO_4_ electrolytes with and without Gly-Gly-20 at 200 mA g^−1^. (**c**) Corresponding charge–discharge curves of MnO_2_//Zn full cells in ZnSO_4_ electrolytes. (**d**) Corresponding charge–discharge curves of MnO_2_//Zn full cells in ZnSO_4_ electrolytes with Gly-Gly-20. (**e**) Illumination of LEDs powered by Zn-MnO_2_ pouch cells in Gly-Gly-20 electrolytes.

To evaluate the performance of the full cell, we selected MnO_2_ [55,56] as the cathode material and pure zinc as the negative electrode and tested the cell in electrolytes with and without Gly-Gly additives. The XRD pattern and SEM images of the MnO_2_ material can be found in Figures S6 and S7. The MnO_2_ material belongs to the α-phase and corresponds perfectly with the standard card (JCPDS 44-0141). Additionally, it can be seen that MnO_2_ exhibits a porous microsphere morphology with a sphere size of about 3 µm. Cyclic voltammetry curves in Figure 4a show that the redox peaks of the full cell with Gly-Gly additives are similar to those without Gly-Gly additives, indicating reversible and unaffected electrochemical processes; both pairs of redox peaks correspond to a two-step reverse redox/oxidation process between MnO_2_ and MnOOH which is ascribed to the H^+^ or Zn^2+^ insertion/extraction reaction from the MnO_2_ cathode. Impressively, better redox reaction kinetic behaviors were gained in the Gly-Gly electrolyte due to less polarization reflecting relatively small potential differences between the two pairs of redox peaks. Furthermore, the Gly-Gly additive improves the cycling performance of the full cell (Figure 4b). After 100 cycles, the full cell with Gly-Gly additives maintains a specific capacity of 206.5 mAh g^−1^, corresponding to 70% capacity retention. By contrast, the specific capacity of the cell with a pure ZnSO_4_ electrolyte rapidly decreases. Moreover, the Gly-Gly additive enabled stable rate performances of the full cells and better cycling reversibility under different charge–discharge rates (Figure S8). According to Figure 4c,d, the introduction of Gly-Gly in the full cell did not lead to a change in the redox reaction mechanism and was capable of increasing the specific capacity of the full cell; this is consistent with the results of CV. These findings highlight the ability of Gly-Gly to establish a stable and adaptive interfacial layer, inhibit side reactions, and significantly contribute to the cycle life of the MnO_2_//Zn full battery. The practical value of the Gly-Gly-20 electrolyte was further demonstrated by assembling a pouch cell battery that successfully lit up an LED light (Figure 4e).

## 3. Experimental Section

### 3.1. Materials and Chemicals

All reagents and materials used in this study were obtained commercially without further purification. Zinc sulfate heptahydrate (ZnSO_4_·7H_2_O, purity 99%), manganese sulfate monohydrate (MnSO_4_·H_2_O, purity 99%), anhydrous ethanol (purity 99.5%), and glycylglycine (Gly-Gly, purity 99%) were sourced from Aladdin Reagent. Zn foil and Ti foil were purchased from Qingyuan Metal Materials. The 7 × 10 cm aluminum–plastic film, 0.1 × 5 × 50 aluminum lugs, and LED light plates were procured from Kolod.

### 3.2. Electrolyte Preparation

The 2M ZnSO_4_ electrolyte was prepared by dissolving zinc sulfate heptahydrate in deionized water. Different amounts of glycylglycine (Gly-Gly) were added to the 2M ZnSO_4_ electrolyte to obtain the Gly-Gly-x (x = 5, 10, 15, 20, 25, 30 g/L) electrolyte, respectively.

### 3.3. Synthesis of MnCO_3_ Microspheres

MnCO_3_ microspheres were synthesized using a simple precipitation method. Initially, 0.507 g of MnSO_4_·H_2_O was dissolved in 70 mL of deionized water, resulting in a clear solution designated as “solution A”. Simultaneously, an equal volume of deionized water was added to another container, and 2.52 g of NaHCO_3_ was dissolved in it, forming “solution B”. Next, 10 mL of ethanol and “solution B” were added to “solution A” while continuously stirring. The addition of these substances resulted in the formation of a visible white precipitate. Subsequently, the suspension was stirred continuously for 3 h at room temperature. The precipitate was then washed three times via centrifugation, using deionized water and alcohol. Finally, the obtained white precipitate was dried in an oven at 60 °C to yield MnCO_3_ powder.

### 3.4. Synthesis of Porous MnO_2_

The MnCO_3_ powder that was produced was transferred into a crucible and placed inside a muffle furnace, where it underwent sintering in an air atmosphere at a temperature of 400 °C for a duration of 5 h. During this process, the MnCO_3_ underwent decomposition, leading to the release of carbon dioxide gas. As a result, the resulting MnO_2_ exhibited a porous structure.

### 3.5. Preparation of MnO_2_ Electrode

MnO_2_, Super P, and PVDF were combined at a mass ratio of 7:2:1. To achieve a homogeneous slurry, an appropriate amount of the *N*-methylpyrrolidone (NMP) solvent was added. This slurry was then coated onto titanium foil using a 100 μm squeegee. The coated film was subsequently dried at 70 °C.

### 3.6. Battery Assembly

CR2032 coin cells were utilized in conducting symmetrical cell, half-cell, and full-cell tests. Zinc foil and peptide foil were cut into 1 cm diameter discs for the cells, with Whatman glass fiber serving as the separator. The cells were assembled at room temperature under an air environment. In the case of pouch cell preparation, a 5 × 4 cm zinc foil and MnO_2_ electrode sheet were cut, separated, assembled according to zinc/fiberglass/manganese dioxide, and wrapped with a layer of the Whatman glass fiber sheet. The cell was then injected with the electrolyte, placed into a 7 × 10 cm flat pocket of aluminum–plastic film, and subjected to pressure and heat sealing. Then, two pouch cells were connected in series and placed inside a new 7 × 10 cm aluminum–plastic film pocket.

### 3.7. Material Characterization

The pH of the electrolyte was determined using a Rigaku PHS-3C pH meter. The surface morphology and corresponding elemental analysis were obtained using a Tescan Mira scanning electron microscope (SEM, Tescan, Brno, Czech Republic). The crystal structures were analyzed using X-ray diffraction (XRD, SmartLab SE, Rigaku, Yamanashi, Japan) with Cu Kα (α = 1.5406 Å) as the radiation source. Fourier transform infrared (FTIR) spectroscopy was performed using a Thermo Scientific iN10 instrument (Thermo Scientific, Waltham, MA, USA). X-ray photoelectron spectroscopy (XPS) was conducted using a Thermo Scientific Kalpha spectrometer (Thermo Scientific, Waltham, MA, USA).

### 3.8. Electrochemical Testing

A LAND-CT3002A (Wuhan LAND Electronic Co., Ltd., Wuhan, China) charge/discharge tester was employed to conduct the constant current charge/discharge testing of the battery. For cyclic voltammetry (CV), electrochemical impedance spectroscopy (EIS), Tafel curve, and chronoamperometry (CA) tests, the symmetrical electrode was employed using a Tatsuwa CHI604E (CH Instruments Co., Ltd., Shang Hai, China) electrochemical workstation. A three-electrode system consisting of titanium foil, zinc foil, and Ag/AgCl electrodes was employed for linear sweep voltammetry (LSV).

### 3.9. DFT Calculation

First-principles calculations based on density functional theory were performed via the Vienna ab-initio simulation package (VASP, 6.1.0). The binding energy (*E_bind_*) of the configurationwas calculated using the following equation.

$$E_{bind}=E_{AB}-\left(E_{A}+E_{B}\right)$$

In the above equation, *E_A_, E_B_*, and *E_AB_* represent the energies of A (Zn^2+^), B (single polymer), and the complex energy, respectively. A negative value for *E_bind_* indicates an exothermic reaction, with a higher negative value indicating a stronger interaction. This stronger interaction corresponds to a greater release of heat and a more stable product.

## 4. Conclusions

In conclusion, we propose an electrolyte optimization strategy for the ZnSO_4_ electrolyte by introducing a low-cost, eco-friendly electrolyte additive of Gly-Gly, which can thus easily enhance the performance of AZIBs. Through theoretical calculations and spectral characterization, we explored the synergistic solvation and interfacial regulation mechanism of Gly-Gly. The adsorption of Gly-Gly molecules onto the zinc cell surface forms a stable and adaptive interfacial layer. This layer regulates the solvation structure of zinc ions, inhibits the reaction between activated water and zinc, and anchors onto the surface of zinc electrodes. As a result, it effectively suppresses the side reactions of zinc electrodes. Finally, with a modest amount of the Gly-Gly additive added to the ZnSO_4_ aqueous electrolyte, highly stable half and full cells were eventually produced. We think that this study opens up new possibilities for Zn anode stabilization and facilitates the development of next-generation electrolytes for energy storage devices.

## Data Availability

The authors confirm that most of the data supporting the findings of this study are available within the article and its supplementary material. Raw data are available from the corresponding author (T.Z. (Tong Zhang)) on request.

## Supplementary Materials

The following supporting information can be downloaded at: https://www.mdpi.com/article/10.3390/molecules29040874/s1, Figure S1: pH of ZnSO_4_ electrolyte with different Gly-Gly concentrations; Figure S2. Morphological analysis of Zinc in (a) pure ZnSO_4_ electrolyte and (b) Gly-Gly-20 electrolyte after 5 Days; Figure S3. The EIS results of Zn-Zn symmetric cells after 10 and 50 cycles in ZnSO_4_ electrolytes without/with Gly-Gly additive; Figure S4. Surface morphology and EDS image of zinc electrodes in (a) pure ZnSO_4_ electrolyte and (b) Gly-Gly-20 electrolyte symmetric cells cycled for 20 h at 1 mA cm^−2^, 1 mAh cm^−2^; Figure S5. SEM image of zinc electrode cycled in Gly-Gly-20 electrolyte at 1 mA cm^−2^, 1 mAh cm^−2^ for 800 h; Figure S6. XRD pattern of MnO_2_; Figure S7. SEM Images of (a) MnO_2_ powder with porous structure, (b) MnO_2_ Electrode after 20 h cycling in Gly-Gly-20 Electrolyte; Figure S8. Rate performances of Zn//MnO_2_ full battery in Electrolyte with/without Gly-Gly-20; Table S1. Element Signal of zinc electrodes in (a) pure ZnSO_4_ electrolyte and (b) Gly-Gly-20 electrolyte symmetric cells cycled for 20 h at 1 mA cm^−2^ 1 mAh cm^−2^.
