# Supplementary material for: Electrolyte Optimization Strategy: Enabling Stable and Eco-Friendly Zinc Adaptive Interfacial Layer in Zinc Ion Batteries
Source: Molecules. 2024 Feb 16;29(4):874. doi: 10.3390/molecules29040874 (PMC10892866; doi:10.3390/molecules29040874)
Supplement: Supplementary file 1 [file molecules-29-00874-s001.zip › molecules-2832337-supplementary.pdf]

Supporting information

Electrolyte Optimization Strategy: Enabling Stable and Eco-friendly Zinc Adaptive Interfacial Layer in Zinc Ion Batteries

Bozhong Cao [1,†], Chunyan Xu [2,†], Bingchun Jiang [1], Biao Jin [1], Jincheng Zhang [1], Lei Ling [1], Yusheng Lu [1], Tianyu Zou [1] and Tong Zhang [1,*]

1. College of Mechanical and Electrical Engineering, Guangdong University of Science and Technology, Dongguan 523000, China

2. Institute for Interdisciplinary Quantum Information Technology, Jilin Engineering Normal University, Changchun 130052, China

† These authors contributed to the work equally and should be regarded as co-first authors.

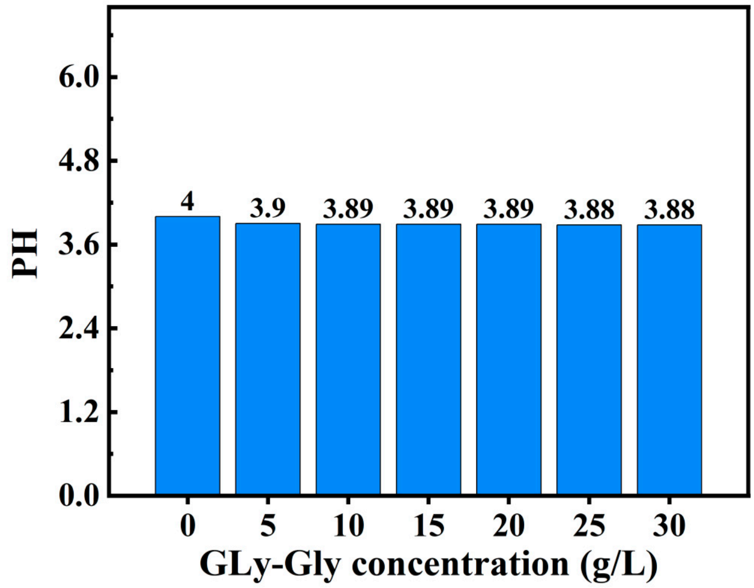

Figure S1. pH of $ZnSO_4$ electrolyte with different Gly-Gly concentrations.

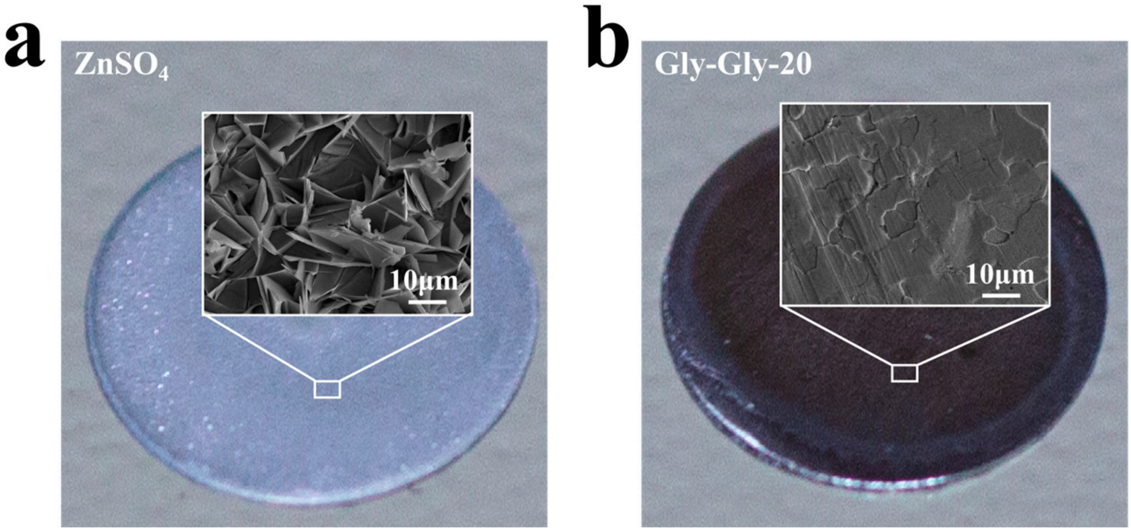

Figure S2. Morphological analysis of Zinc in (**a**) pure $ZnSO_4$ electrolyte and (**b**) Gly-Gly-20 electrolyte after 5 Days.

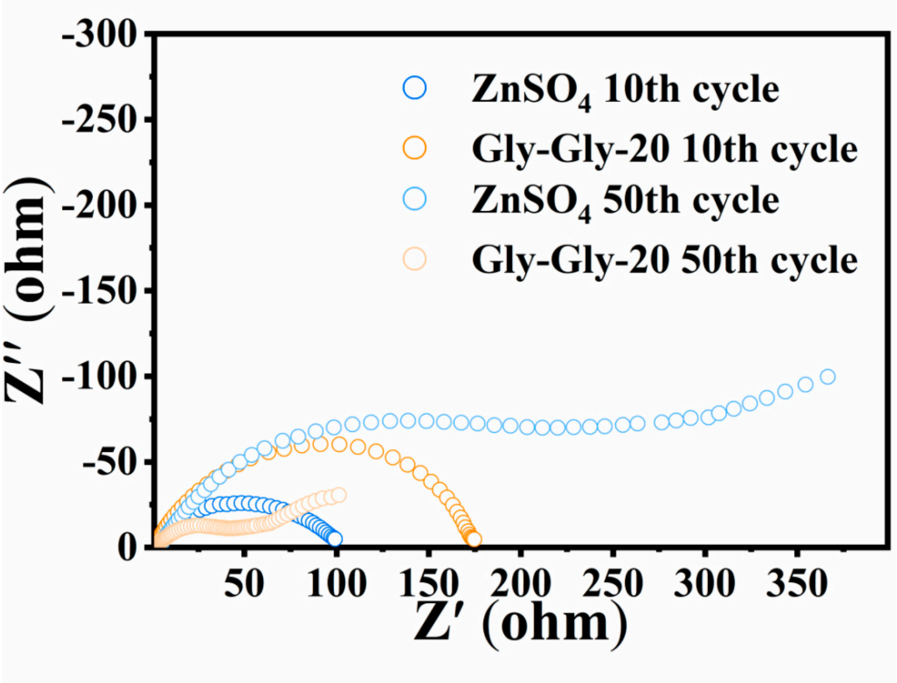

Figure S3. The EIS results of Zn-Zn symmetric cells after 10 and 50 cycling in $ZnSO_4$ electrolytes without/with Gly-Gly additive.

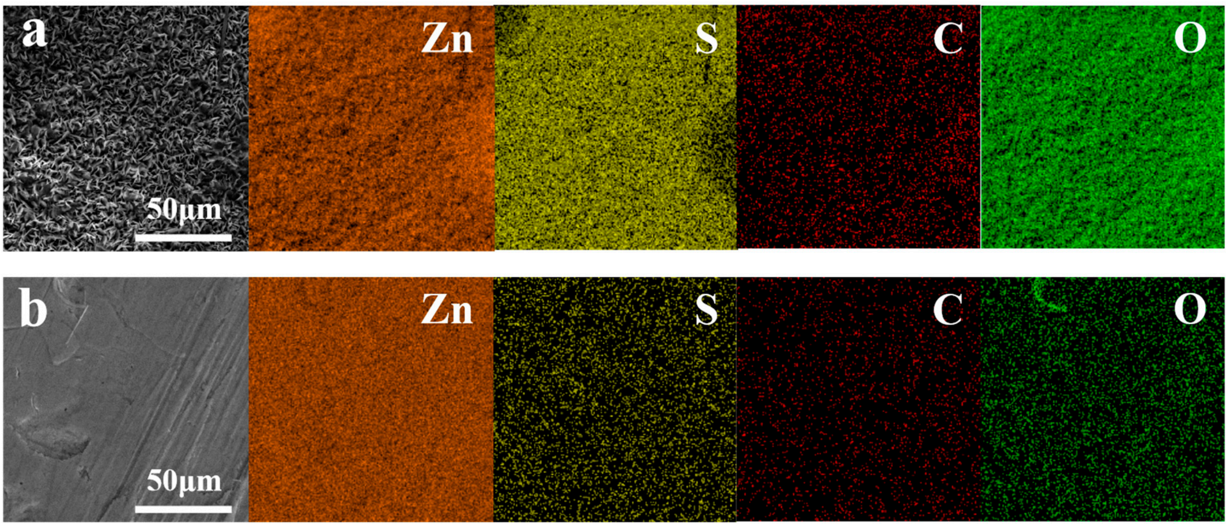

Figure S4. Surface morphology and EDS image of zinc electrodes in (**a**) pure $ZnSO_4$ electrolyte and (**b**) Gly-Gly-20 electrolyte symmetric cells cycled for 20h at 1mA cm^{-2} 1mAh cm^{-2}.

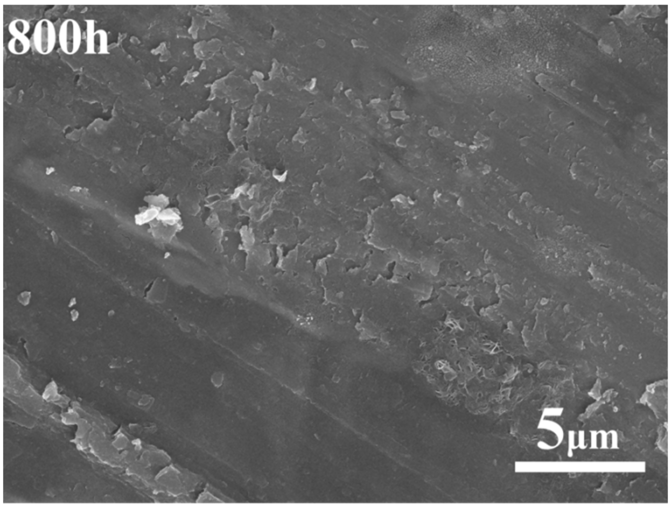

Figure S5. SEM image of zinc electrode cycled in Gly-Gly-20 electrolyte at 1 mA cm^{-2} 1 mAh cm^{-2} for 800h.

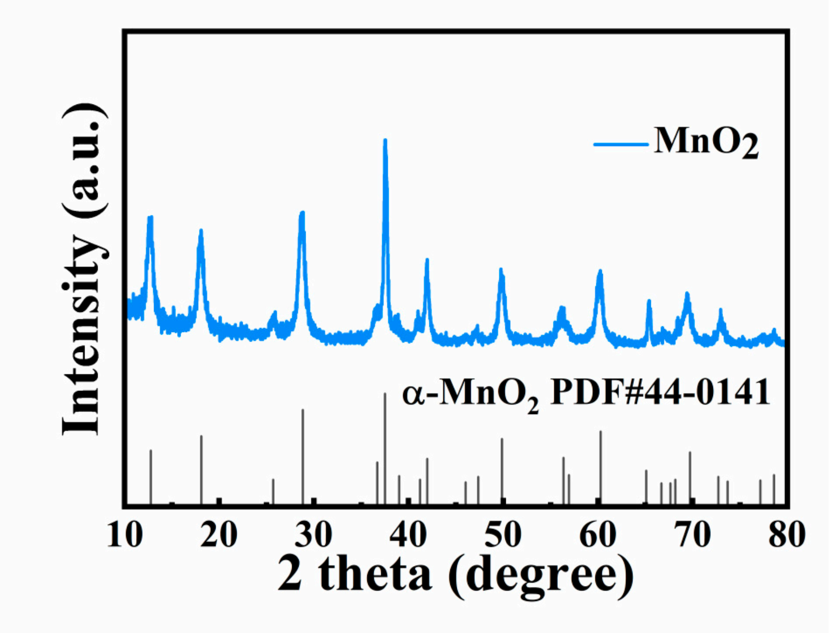

Figure S6. XRD pattern of MnO_2.

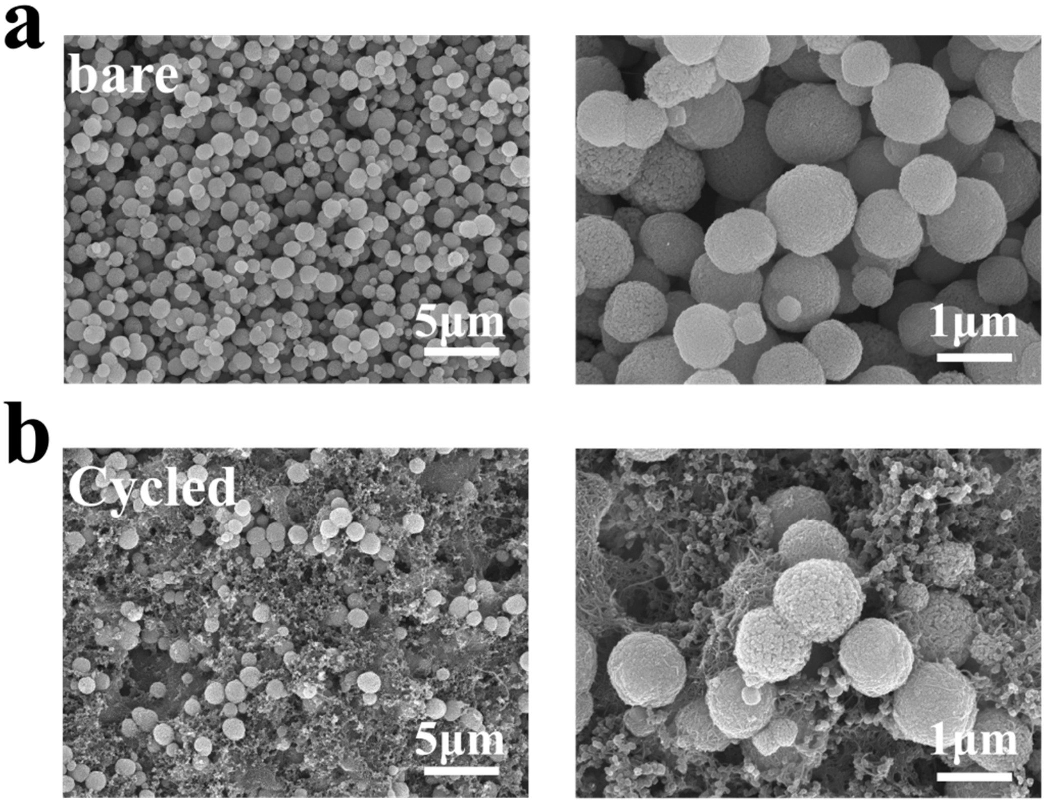

Figure S7. SEM Images of (**a**) MnO_2 powder with porous structure, (**b**) MnO_2 Electrode after 20h cycling in Gly-Gly-20 Electrolyte.

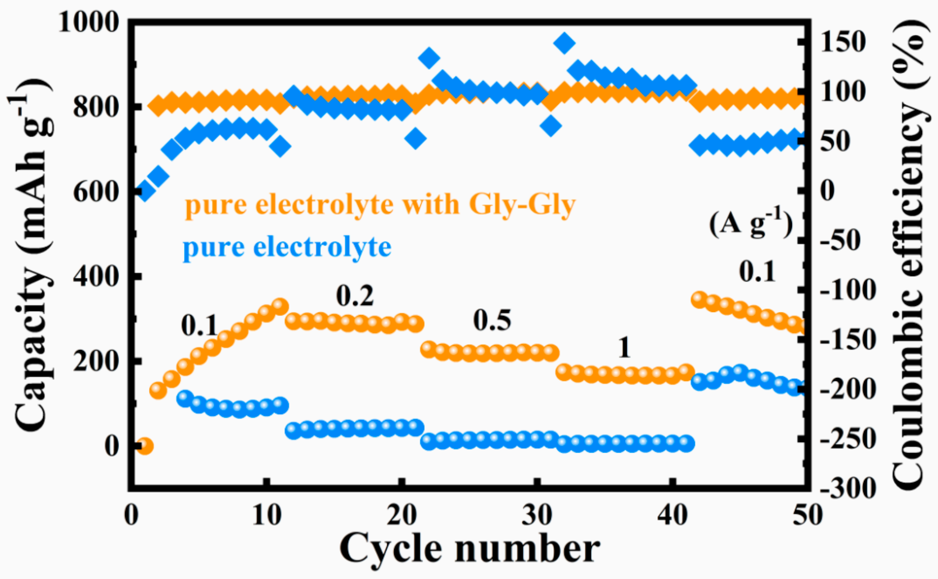

Figure S8. Rate performances of Zn//MnO_2 full battery in Electrolyte with/without Gly-Gly-20.

Table S1. Element Signal of zinc electrodes in pure $ZnSO_4$ electrolyte and Gly-Gly-20 electrolyte symmetric cells cycled for 20 h at 1 mA cm^{-2} 1 mAh cm^{-2}.

**electrolyte**	**Zn Weight %**	**S Weight %**	**C Weight %**	**O Weight %**
$ZnSO_4$	52.64	31.00	8.55	7.82
Gly-Gly-20	94.21	4.44	1.30	0.04
